# Supplementary material for: Inhibition of CDK12 elevates cancer cell dependence on P-TEFb by stimulation of RNA polymerase II pause release
Source: Nucleic Acids Res. 2023 Oct 9;51(20):10970–91. doi: 10.1093/nar/gkad792 (PMC10639066; doi:10.1093/nar/gkad792)
Supplement: gkad792_Supplemental_Files [file gkad792_supplemental_files.zip › Supplementary Data.pdf]

Supplementary Data

Inhibition of CDK12 elevates cancer cell dependence on P-TEFb by stimulation of RNA polymerase II pause release

Zhijia Wang,[1] Samu V. Himanen,[2] Heidi M. Haikala,[3,4] Caroline C. Friedel,[5] Anniina Vihervaara,[2] Matjaž Barborič [1,*]

[1]Department of Biochemistry and Developmental Biology, University of Helsinki, Helsinki FIN-00014, Finland

[2]Department of Gene Technology, KTH Royal Institute of Technology, Science for Life Laboratory, Stockholm, Sweden

[3]Translational Immunology Research Program (TRIMM), Research Programs Unit, Faculty of Medicine, University of Helsinki, Helsinki FIN-00014, Finland

[4]iCAN Digital Precision Cancer Medicine Flagship, University of Helsinki, Helsinki FIN-00014, Finland

[5]Institute of Informatics, Ludwig-Maximilians-Universität München, 80333 Munich, Germany

[*]Correspondence: matjaz.barboric@helsinki.fi

Contents:

1. Supplementary Figures S1-S7

2. Supplementary Table S1

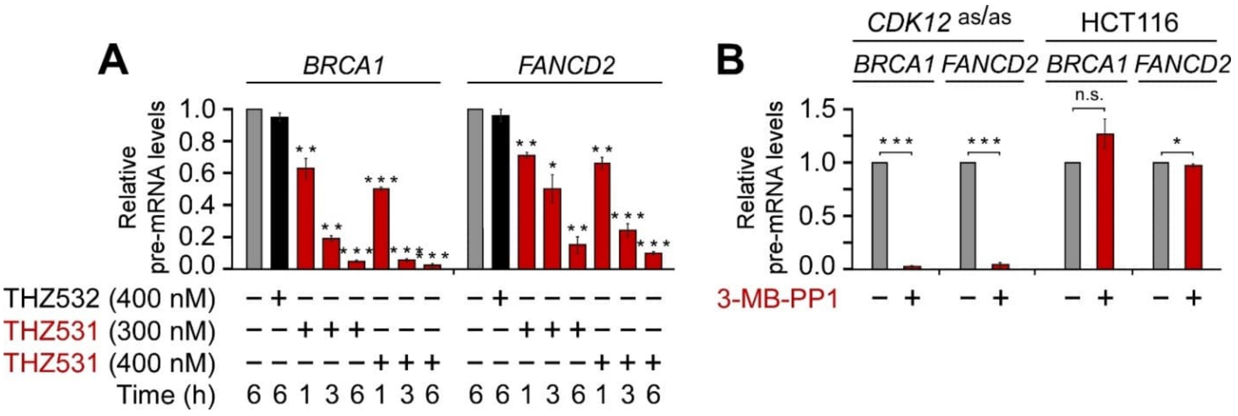

Supplementary Figure S1. Inhibition of CDK12 triggers the release of P-TEFb from the inhibitory 7SK snRNP complex.

(A) HCT116 cells were treated with DMSO (-), THZ532 and two different doses of THZ531 as indicated for the indicated duration prior to quantifying pre-mRNA levels of *BRCA1* and *FANCD2* with RT-qPCR. Results normalized to the levels of GAPDH mRNA and DMSO-treated cells are presented as the mean ± s.e.m. (n = 3). *, P < 0.05; **, P < 0.01; ***, P < 0.001, determined by Student's *t* test.

(B) HCT116 cell lines were treated with DMSO (-) and 3-MB-PP1 (5 μM) as indicated for 3 h prior to quantifying pre-mRNA levels of *BRCA1* and *FANCD2* with RT-qPCR. Results normalized to the levels of GAPDH mRNA and DMSO-treated cells are presented as the mean ± s.e.m. (n = 3). *, P < 0.05; ***, P < 0.001; n.s., non-significant, determined by Student's *t* test.

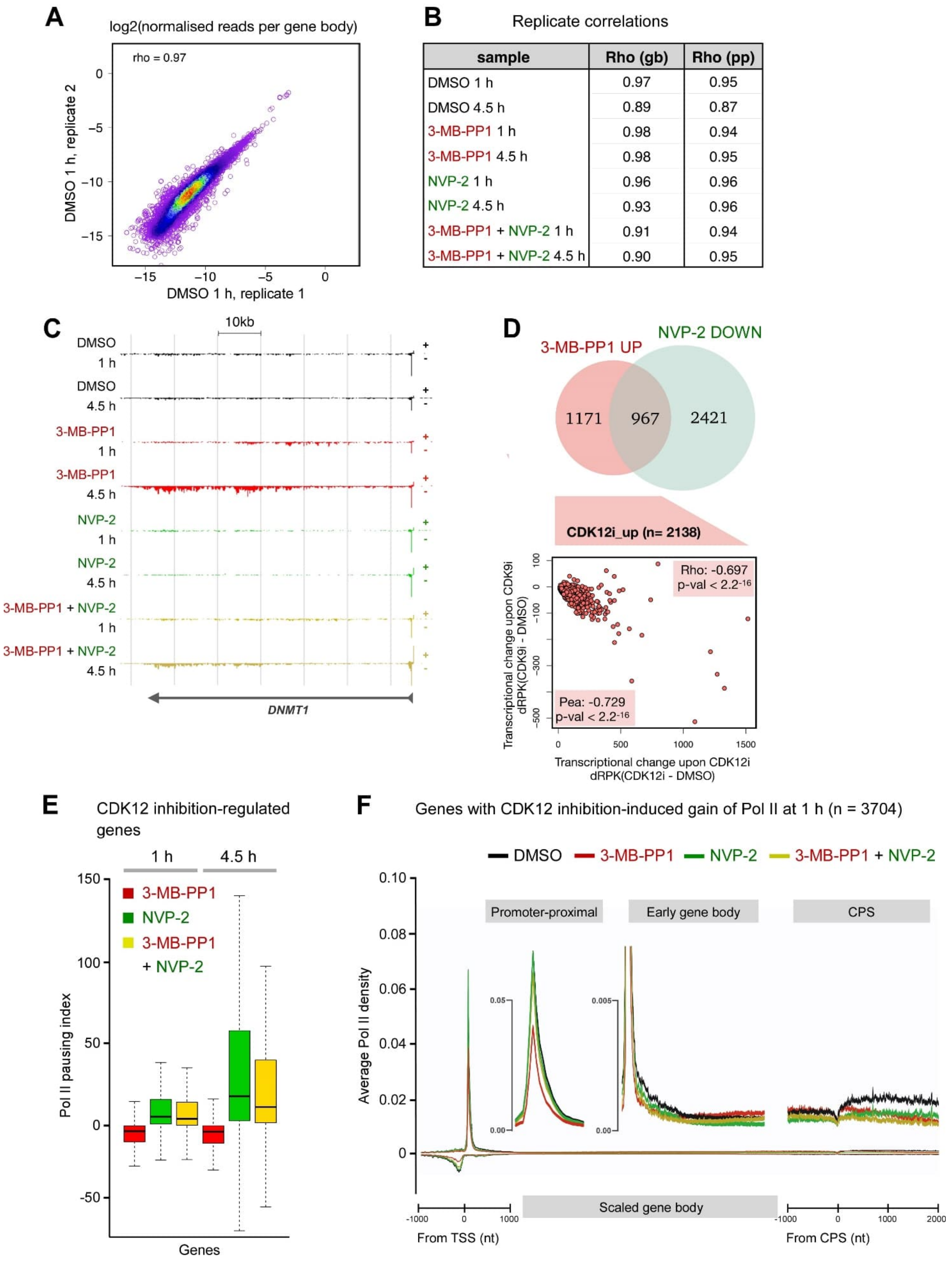

Supplementary Figure S2. Transcriptional induction in CDK12-inhibited cells occurs by P-TEFb-stimulated Pol II pause release.

(A) Representative correlation plot comparing PRO-seq replicates (n = 2) of HCT116 *CDK12* $^{as/as}$ cells treated with DMSO for 1 h. The x- and y-axes show log2 count of normalized PRO-seq reads across the gene bodies from +500 nt from the TSS to -500 nt from the TES.

(B) Correlations between the PRO-seq replicates (n = 2) of HCT116 *CDK12* $^{as/as}$ cells treated with DMSO, 3-MB-PP1 (5 μM) and NVP-2 (10 nM) alone and in combination as indicated for 1 h and 4.5 h across promoter-proximal region (pp; -250 nt to +250 nt from the TSS) and gene body (gb) as defined in (A). Rho indicates Spearman's rank correlation.

(C) Density of engaged Pol II across *DNMT1* gene at 1 h or 4.5 h from PRO-seq replicates (n = 2) of HCT116 *CDK12* $^{as/as}$ cells treated with DMSO, 3-MB-PP1 (5 μM) and NVP-2 (10 nM) alone and in combination as indicated. Please note that the wave of transcriptional induction upon inhibition of CDK12 has proceeded 40 kb at 1 h, and through the entire gene at 4.5 h of the treatment.

(D) Venn diagram (top) and correlation plot (bottom) comparisons between CDK12-induced and P-TEFb-repressed genes at 4.5 h of the PRO-seq replicated (n = 2) of HCT116 *CDK12* $^{as/as}$ cells treated with 3-MB-PP1 (5 μM) and NVP-2 (10 nM), respectively. Rho and Pea indicate Spearman's and Pearson's rank correlations, respectively.

(E) Change in pausing index of CDK12 inhibition-regulated genes derived from PRO-seq replicates (n = 2) of HCT116 *CDK12* $^{as/as}$ cells treated with 3-MB-PP1 (5 μM) and NVP-2 (10 nM) alone and in combination as indicated for 1 h or 4.5 h compared to DMSO. Median pausing index for each group is indicated.

(F) Average density of engaged Pol II at genes with CDK12 inhibition-induced gain of Pol II at 1 h from PRO-seq experiments (n = 2) of (A). The promoter-proximal region is measured as a linear scale from –1000 to +1000 nucleotide from the TSS, gene body scaled into 50 bins per gene, and end of the gene as a linear scale from –1000 to + 2000 nucleotide from the CPS. The insets show magnified promoter-proximal, early gene body and CPS gene regions. TSS, transcription start site. CPS, cleavage and polyadenylation site. nt, nucleotide.

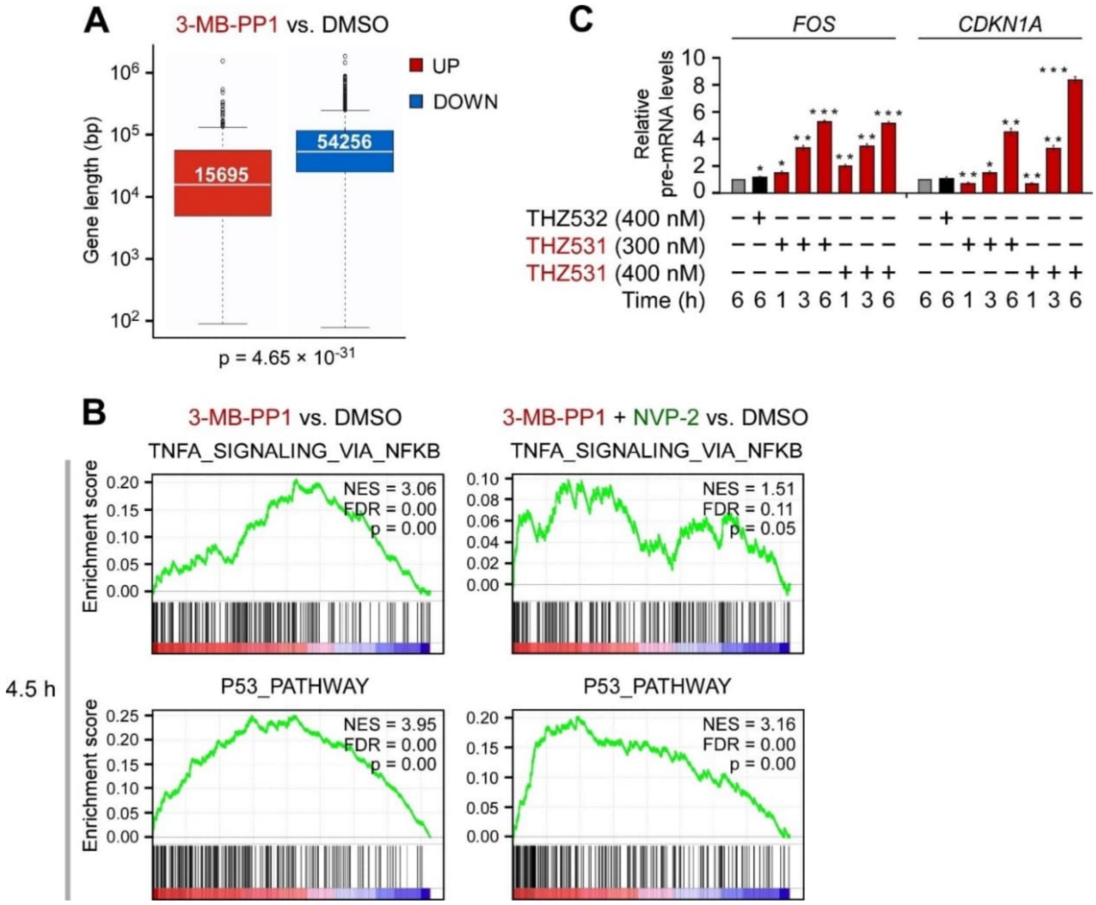

Supplementary Figure S3. Inhibition of CDK12 induces gene expression downstream of key cancer pathways.

(A) Boxplot indicating distribution of gene lengths for the induced (UP) and down-regulated (DOWN) protein-coding genes from RNA-seq experiments (n = 3) of serum-synchronized HCT116 *CDK12* $^{as/as}$ cells treated for 4.5 h with DMSO or 3-MB-PP1 (5 μM). Median gene length for each group and p-value are indicated. bp, base pair.

(B) Enrichment plots of the top NF-κB and p53 pathway gene sets of Figure 3B from the GSEA of transcription changes of protein-coding genes obtained from PRO-seq experiments (n = 2) of HCT116 *CDK12* $^{as/as}$ cells treated with DMSO, 3-MB-PP1 (5 μM) and NVP-2 (10 nM) as indicated for 4.5 h. NES, normalized enrichment score; positive values indicate enrichment among induced genes, negative values enrichment among down-regulated genes. FDR, false discovery rate.

(C) HCT116 cells were treated with DMSO, THZ532 and two different doses of THZ531 as indicated for the indicated duration prior to quantifying pre-mRNA levels of *FOS* and *CDKN1A* with RT-qPCR. Results normalized to the levels of GAPDH mRNA and DMSO-treated cells are presented as the mean ± s.e.m. (n = 3). *, P < 0.05; **, P < 0.01; ***, P < 0.001, determined by Student's *t* test.

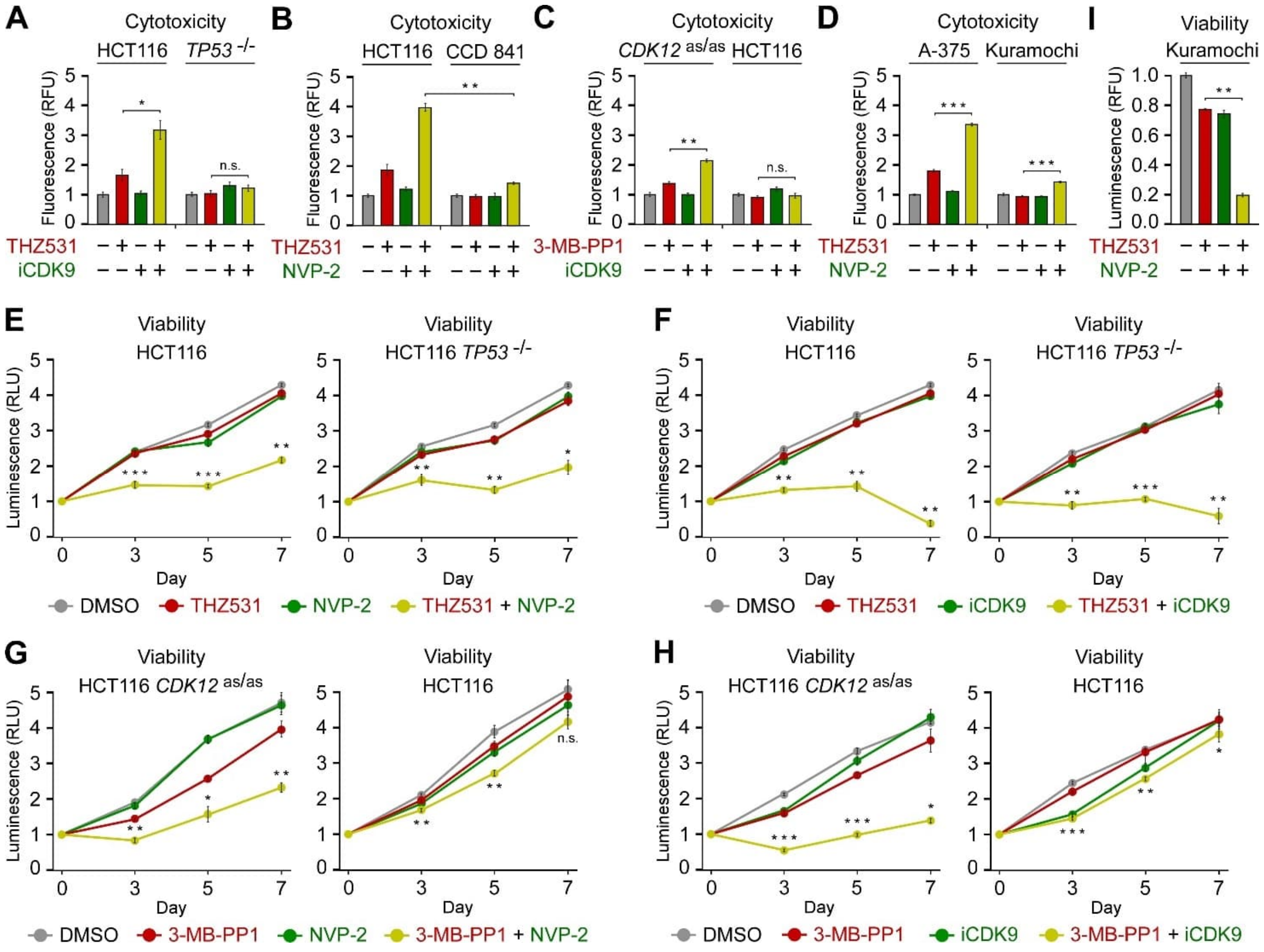

Supplementary Figure S4. Co-targeting of CDK12 and P-TEFb decreases viability of cancer cells.

(A-D) Cytotoxicity of the indicated cell lines treated with DMSO (-), THZ531 (200 nM), 3-MB-PP1 (5 μM) and iCDK9 (25 nM) alone and in combination as indicated for 48 h measured using CellTox Green Cytotoxicity Assay. Results are presented as fluorescence values relative to the values of DMSO-treated cells and plotted as the mean ± s.e.m. (n = 3). *, P < 0.05; **, P < 0.01; ***, P < 0.001; n.s., non-significant, determined by Student's *t* test.

(E,F) Viability of HCT116 cell lines treated with DMSO, THZ531 (50 nM), NVP-2 (2.5 nM) and iCDK9 (25 nM) alone and in combination as indicated. Results obtained at the time points indicated below the graphs using CellTiter-Glo 2.0 Cell Viability Assay are presented as luminescence values relative to the values at Day 0 and plotted as the mean ± s.e.m. (n = 3). *, P < 0.05; **, P < 0.01; ***, P < 0.001, determined by Student's *t* test using THZ531 and THZ531 + NVP-2 (E), and THZ531 and THZ531 + iCDK9 (F) data sets.

(G,H) Viability of HCT116 cell lines treated with DMSO, 3-MB-PP1 (2.5 μM), NVP-2 (1.25 nM) and iCDK9 (5 nM) alone and in combination as indicated. Results obtained at the time points indicated below the graphs using CellTiter-Glo 2.0 Cell Viability Assay are presented as luminescence values relative to the values at Day 0 and plotted as the mean ± s.e.m. (n = 3). *, P < 0.05; **, P < 0.01, ***, P < 0.001, determined by Student's *t* test using 3-MB-PP1 and 3-MB-PP1 + NVP-2 (G), and 3-MB-PP1 and 3-MB-PP1 + iCDK9 (H) data sets.

(I) Viability of Kuramochi cells treated with DMSO, THZ531 (50 nM) and NVP-2 (2.5 nM) alone and in combination as indicated. Results obtained at the time points indicated below the graphs using CellTiter-Glo 2.0 Cell Viability Assay are presented as luminescence values relative to the values at Day 0 and plotted as the mean ± s.e.m. (n = 3). **, P < 0.01, determined by Student's *t* test using THZ531 and THZ531 + NVP-2 data sets.

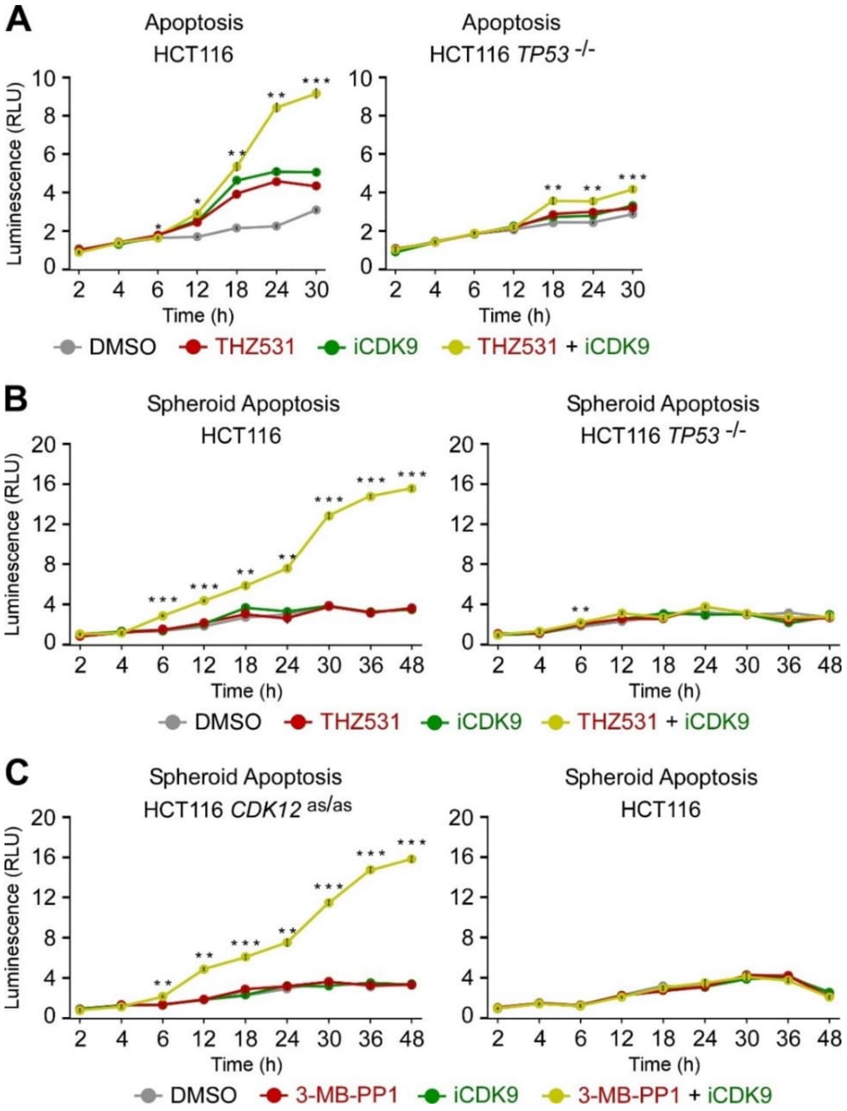

Supplementary Figure S5. Co-targeting of CDK12 and P-TEFb stimulates apoptosis of HCT116 cells.

(A) Apoptosis of HCT116 cell lines treated with DMSO, THZ531 (100 nM) and iCDK9 (25 nM) alone and in combination as indicated. Results obtained at the time points indicated below the graphs using RealTime-Glo Annexin V Apoptosis and Necrosis Assay are presented as luminescence values relative to the values of DMSO-treated cells at 2 h and plotted as the mean ± s.e.m. (n = 3). *, P < 0.05; **, P < 0.01; ***, P < 0.001, determined by Student's *t* test using THZ531 and THZ531 + iCDK9 data sets.

(B,C) Apoptosis of HCT116 cell line spheroid cultures treated with DMSO, THZ531 (100 nM), 3-MB-PP1 (5 μM) and iCDK9 (25 nM) alone and in combination as indicated. Spheroids were formed for 48 h prior to the treatments. Results obtained at the time points indicated below the graphs using RealTime-Glo Annexin V Apoptosis and Necrosis Assay are presented as luminescence values relative to the values of DMSO-treated cells at 2 h and plotted as the mean ± s.e.m. (n = 3). **, P < 0.01; ***, P < 0.001, determined by Student's *t* test using THZ531 and THZ531 + iCDK9 (B), and 3-MB-PP1 and 3-MB-PP1 + iCDK9 (C) data sets.

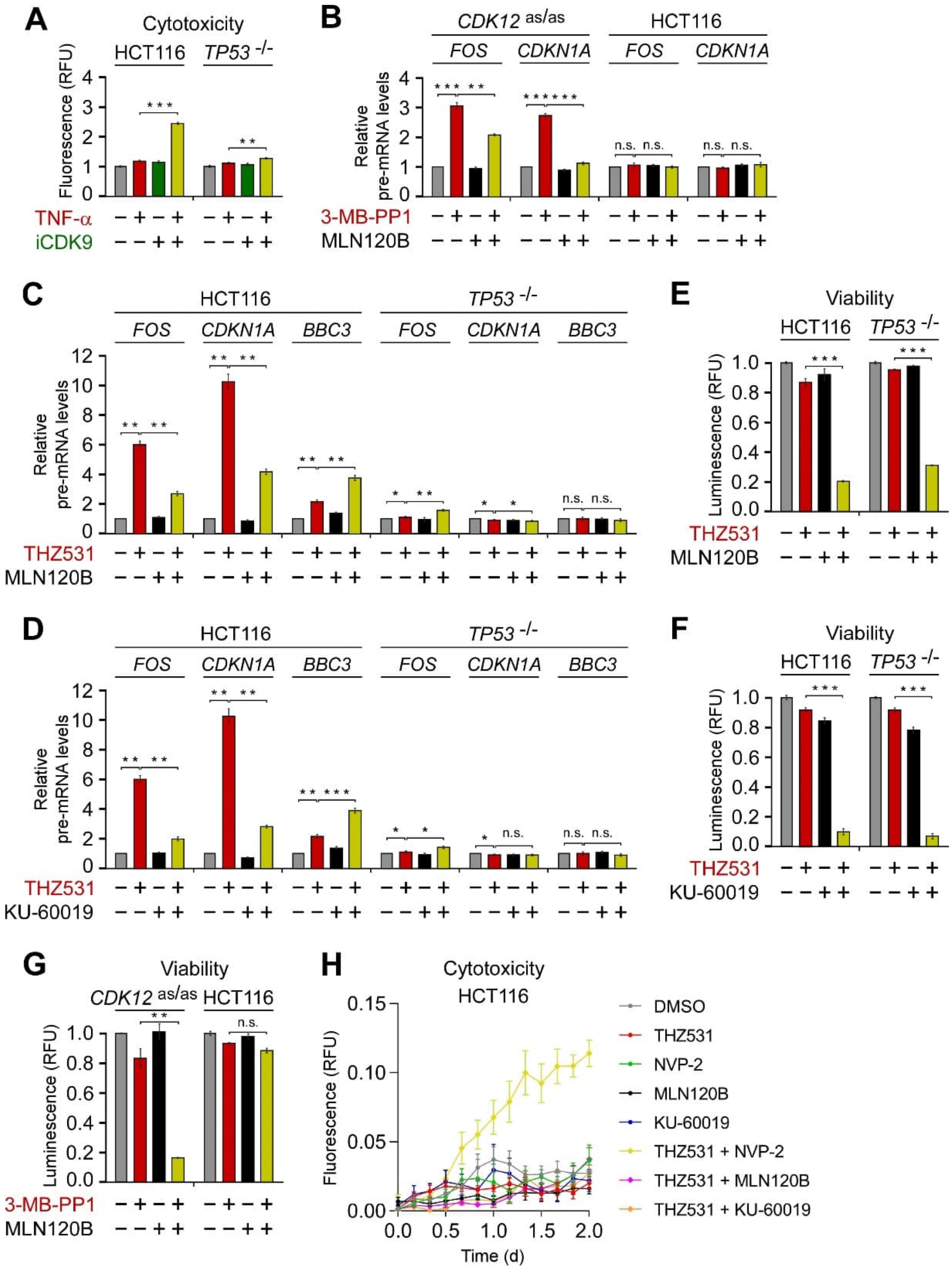

Supplementary Figure S6. Inhibition of CDK12 renders cancer cells dependent on the NF-κB pathway.

(A) Cytotoxicity of HCT116 cell lines treated with DMSO (-), TNF-α (20 ng/ml) and iCDK9 (25 nM) alone and in combination as indicated for 48 h measured using CellTox Green Cytotoxicity Assay. Results are presented as fluorescence values relative to the values of DMSO-treated cells and plotted as the mean ± s.e.m. (n = 3). **, $P < 0.01$; ***, $P < 0.001$, determined by Student's *t* test.

(B-D) HCT116 cell lines were treated with DMSO (-), THZ531 (400 nM), 3-MB-PP1 (5 μM), MLN120B (20 μM) and KU-60019 (5 μM) alone and in combination as indicated for 3 h (B) or 12 h (C, D) prior to quantifying pre-mRNA levels of *FOS*, *CDKN1A* and *BBC3* with RT-qPCR. Results normalized to the levels of GAPDH mRNA and DMSO-treated cells are presented as the mean ± s.e.m. (n = 3). *, $P < 0.05$; **, $P < 0.01$; ***, $P < 0.001$, n.s., non-significant, determined by Student's t test.

(E-G) Viability of HCT116 cell lines treated with DMSO, THZ531 (50 nM), 3-MB-PP1 (2.5 μM), MLN120B (2.5 μM in E; 5 μM in F) and KU-60019 (50 nM) alone and in combination as indicated for seven days measured using CellTiter-Glo 2.0 Cell Viability Assay. Results are presented as luminescence values relative to the values of DMSO-treated cells and plotted as the mean ± s.e.m. (n = 3). **, $P < 0.01$; ***, $P < 0.001$; n.s., non-significant, determined by Student's t test.

(H) Cytotoxicity of HCT116 cell lines treated with DMSO, THZ531 (50 nM), NVP-2 (1.25 nM), MLN120B (2.5 μM) and KU-60019 (50 nM) alone and in combination as indicated for 48 h measured using CellTox Green Cytotoxicity Assay. Results are presented as fluorescence values and plotted as the mean ± s.e.m. (n = 3).

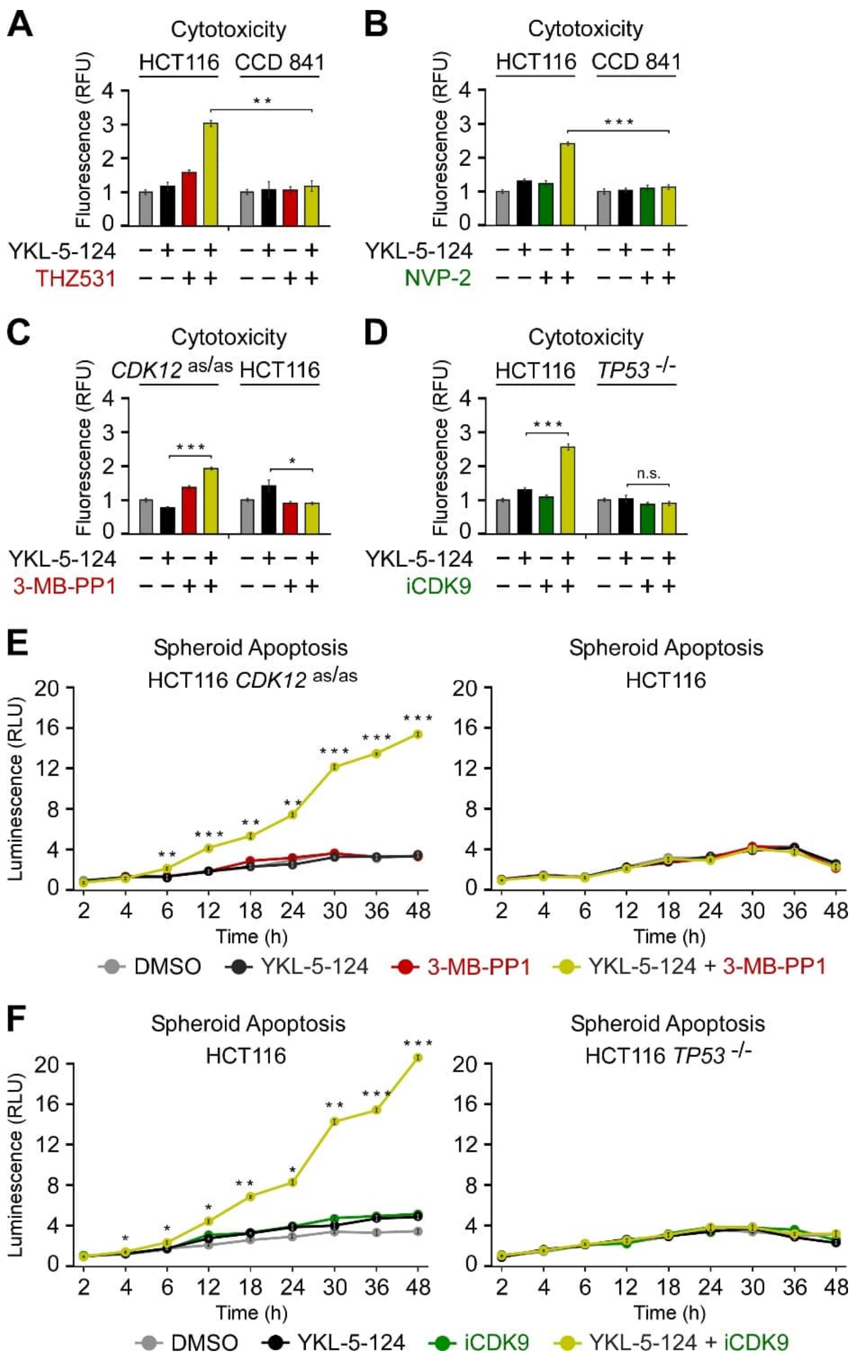

Supplementary Figure S7. Co-targeting of CDK7 and either CDK12 or P-TEFb stimulates apoptosis of HCT116 cells.

(A-D) Cytotoxicity of the indicated cell lines treated with DMSO (-), YKL-5-124 (100 nM), THZ531 (200 nM), 3-MB-PP1 (5 μM), NVP-2 (10 nM) and iCDK9 (25 nM) alone and in combination as indicated for 48 h measured using CellTox Green Cytotoxicity Assay. Results are presented as fluorescence values relative to the values of DMSO-treated cells and plotted as the mean ± s.e.m. (n = 3). **, P < 0.01; ***, P < 0.001; n.s., non-significant, determined by Student's *t* test.

(E,F) Apoptosis of HCT116 cell line spheroid cultures treated with DMSO, YKL-5-124 (100 nM), 3-MB-PP1 (5 μM) and iCDK9 (25 nM) alone and in combination as indicated. Spheroids were formed for 48 h prior to the treatments. Results obtained at the time points indicated below the graphs using RealTime-Glo Annexin V Apoptosis and Necrosis Assay are presented as luminescence values relative to the values of DMSO-treated cells at 2 h and plotted as the mean ± s.e.m. (n = 3). *, P < 0.05; **, P < 0.01; ***, P < 0.001, determined by Student's *t* test using YKL-5-124 and YKL-5-124 + 3-MB-PP1 (F), and YKL-5-124 and YKL-5-124 + iCDK9 (F) data sets.

Supplementary Table S1A. Antibodies used in the study.

Antibody	Source	Identifier
Mouse monoclonal anti-CDK9	Santa Cruz Biotechnology	Cat#sc-13130; RRID: AB_627245
Mouse monoclonal anti-p53	Santa Cruz Biotechnology	Cat#sc-126; RRID: AB_628082
Mouse monoclonal anti-p21	Santa Cruz Biotechnology	Cat#sc-53870; RRID: AB_785026
Mouse monoclonal anti-GAPDH	Santa Cruz Biotechnology	Cat#sc-32233; RRID: AB_627679
Rabbit polyclonal anti-CDK12	Cell Signaling Technology	Cat#11973S; RRID: AB_2715688
Goat polyclonal anti-HEXIM1	Everest Biotech	Cat#EB06964; RRID: AB_2118260
Mouse monoclonal anti-CDK7	Cell Signaling Technology	Cat#2916; RRID: AB_2077142

Supplementary Table S1B. Chemicals used in the study.

Chemical	Source	Identifier
NVP-2	MedChemExpress	Cat#HY-12214A
i-CDK9	Qiang Zhou Laboratory (UC Berkeley)	N/A
THZ531	MedChemExpress	Cat#HY-103618
THZ532	Nathanael S. Gray Laboratory (Stanford University)	N/A
3-MB-PP1	Cayman Chemical	Cat#CAY17860
YKL-5-124	Nathanael S. Gray Laboratory (Stanford University)	N/A
TNF-α	Proteintech	Cat#HZ-1014
KU-60019	Selleck Chemicals	Cat#S1570
MLN120B	MedChemExpress	Cat#HY-15473
Triton X-100	Merck	Cat#108603
Propidium iodide	Merck	Cat#537059
RNase A	Thermo Fisher Scientific	Cat#12091021
EDTA-free Protease Inhibitor Cocktail	Merck	Cat#11873580001
Complete Protease Inhibitor Cocktail	Merck	Cat# 11697498001
PhosSTOP Phosphatase Inhibitor Cocktail	Merck	Cat# 4906837001
Phenylmethanesulfonyl fluoride	Merck	Cat# 10837091001
Random hexamers	Thermo Fisher Scientific	Cat#N8080127
TRI Reagent	Merck	Cat#T9424
SUPERase•In RNase Inhibitor (20 U/µL)	Thermo Fisher Scientific	Cat#AM2694
Biotin-11-ATP	Perkin Elmer	Cat# NEL544001EA
Biotin-11-CTP	Perkin Elmer	Cat# NEL542001EA
Biotin-11-GTP	Perkin Elmer	Cat# NEL545001EA
Biotin-11-UTP	Perkin Elmer	Cat# NEL543001EA
TRIzol Reagent	Thermo Fisher Scientific	Cat#15596026
TRIzol LS Reagent	Thermo Fisher Scientific	Cat#0296028
GlycoBlue Coprecipitant (15 mg/mL)	Thermo Fisher Scientific	Cat# AM9516

Supplementary Table S1C. Commercial assays used in the study.

Assay	Source	Identifier
CellTox™ Green Cytotoxicity Assay	Promega	Cat#G8731
CellTiter-Glo® 2.0 Cell Viability Assay	Promega	Cat#G242
RealTime-Glo Annexin V Apoptosis and Necrosis Assay	Promega	Cat#JA1011
NP-40 lysis buffer	Thermo Fisher Scientific	Cat#J60766-AP
M-MLV reverse transcriptase	Thermo Fisher Scientific	Cat#28025-013
Turbo DNA-freeTM kit	Thermo Fisher Scientific	Cat#AM1907
Dynabeads Protein G	Thermo Fisher Scientific	Cat#10004D
FastStart Universal SYBR Green QPCR Master (Rox)	Merck	Cat#4913914001
Mycoplasmacheck detection kit	Eurofins	Cat#50400400
Bio-Spin P-30 Gel Columns, Tris Buffer	Bio-Rad	Cat# 7326231
Dynabeads MyOne Streptavidin C1	Thermo Fisher Scientific	Cat#65002
T4 RNA Ligase 1 (ssRNA Ligase)	New England Biolabs	Cat#M0204S
RNA 5' Pyrophosphohydrolase (RppH)	New England Biolabs	Cat#M0356S
T4 Polynucleotide Kinase	New England Biolabs	Cat# M0201S
SuperScript III Reverse Transcriptase	Thermo Fisher Scientific	Cat# 18080044
Phusion Polymerase	In-house	N/A
Mag-Bind TotalPure NGS	Omega Bio-tek	Cat# M1378-00

Supplementary Table S1D. Cell lines used in the study.

Cell line	Source	Identifier
HCT116 and HCT116 *TP53* $^{-/-}$	Joaquin M. Espinosa Laboratory (University of Colorado)	N/A
HCT116 and HCT116 *CDK12* $^{as/as}$	Dalibor Blazek Laboratory (CEITEC)	N/A
CCD 841 CoN	ATCC	Cat#CRL-1790

Supplementary Table S1E. Software and algorithms used in the study.

Software	Source	Identifier
R	N/A	https://www.R-project.org/
Molecular Signatures Database v6.0	GSEA - Broad Institute (Subramanian et al., 2005)	http://software.broadinstitute.org/gsea/msigdb/index.jsp
dREG	Wang et al., 2019	https://dreg.js2.scigap.org
Bowtie2	Langmead & Salzberg, 2012	https://bowtie-bio.sourceforge.net/bowtie2/index.shtml
Samtools	Li et al., 2009	http://www.htslib.org
Fastp	Chen et al., 2018	https://github.com/OpenGene/fastp
Bedtools	Quinlan Laboratory, University of Utah (Quinlan & Hall, 2010)	https://github.com/arq5x/bedtools2

Supplementary Table S1F. DNA oligonucleotides used in RT-qPCR assay.

Gene	Primer Sequence (5'-3')
FOS	GAGGGAGCTGACTGATACA
	GAGGGATAAAGGAAAGCATAAGA
CDKN1A	TCTGCCTCCCAGAGTATTAG
	GAGTCCTGTGCTGTATTG
GAPDH	CGACCACTTTGTCAAGCTCA
	AGGGGAGATTCAGTGTGGTG

Supplementary Table S1G. Adapter and primer sequences used in PRO-seq.

Adapter/primer	Adapter/primer Sequence (5'-3')
Barcoded VR3 adapter BR15 (3'adapter)	rGrArUrGrUrCrArGrArUrCrGrUrCrGrGrArCrUrGrUrArGrArArCrUrCrUrGrArArCr /3InvdT/
Barcoded VR3 adapter BR17 (3'adapter)	GrGrUrArGrArGrGrArUrCrGrUrCrGrGrArCrUrGrUrArGrArArCrUrCrUrGrArArCr /3InvdT/
Rev5 adapter (5'adapter + 5'UMI)	/5InvddT/CCTTGGCACCCGAGAATTCCANrNrNrNrNrNrC
RP1 primer	AATGATACGGCGACCACCGAGATCTACACGTTCAGAGTTCTACAGTCCGA
RPI-4 primer	CAAGCAGAAGACGGCATACGAGAT TGGTCAGTGACTGGAGTTCCTTGGCACCCGAGAATTCCA
RPI-5 primer	CAAGCAGAAGACGGCATACGAGAT CACTGTGTGACTGGAGTTCCTTGGCACCCGAGAATTCCA
RPI-6 primer	CAAGCAGAAGACGGCATACGAGAT ATTGGCGTGACTGGAGTTCCTTGGCACCCGAGAATTCCA
RPI-7 primer	CAAGCAGAAGACGGCATACGAGAT GATCTGGTGACTGGAGTTCCTTGGCACCCGAGAATTCCA
RPI-8 primer	CAAGCAGAAGACGGCATACGAGAT TCAAGTGTGACTGGAGTTCCTTGGCACCCGAGAATTCCA
RPI-9 primer	CAAGCAGAAGACGGCATACGAGAT CTGATCGTGACTGGAGTTCCTTGGCACCCGAGAATTCCA
